# Avenanthramide Improves Colonic Damage Induced by Food Allergies in Mice through Altering Gut Microbiota and Regulating Hsp70-NF-κB Signaling

**DOI:** 10.3390/nu15040992

**Published:** 2023-02-16

**Authors:** Pan Liu, Mingrui Zhang, Tianyi Liu, Ruixia Mo, Haotian Wang, Gang Zhang, Yi Wu

**Affiliations:** State Key Laboratory of Animal Nutrition, College of Animal Science and Technology, China Agricultural University, Beijing 100193, China

**Keywords:** avenanthramide, colonic injury, food allergy, gut microbiota, butyrate, Hsp70-NF-κB signaling

## Abstract

Food allergies can cause intestinal damage that can exacerbate allergic symptoms, and gut microbiota have been shown to influence allergic development. This study was intended to investigate the effects of Avenanthramide (AVA) on colonic damage induced by food allergy and its mechanism. In Exp. 1, AVA administrations alleviated colonic inflammation in mice challenged with ovalbumin, as shown by decreased concentrations of TNF-α, IL-25 and IL-33. Additionally, the AVA supplementations improved intestinal barrier damage by elevating occludin, ZO-1 and claudin-1 levels. Moreover, AVA inhibited NF-κB phosphorylation and enhanced heat shock protein 70 (Hsp70) expression in the colon. In Exp. 2, apoptozole as a Hsp70 inhibitor was used to explore the Hsp70-NF-κB signaling contribution to AVA function. The AVA additions increased the productions of acetate and butyrate, but decreased propionate. Notably, AVA reduced the colonic abundance of propionate-producing microbes such as Muribaculaceae, but elevated butyrate-producing microbes including *Roseburia*, *Blautia*, and *Lachnospiraceae_NK4A136_group*. Microbial alteration could be responsible for the increased butyrate, and thus the up-regulated Hsp70. However, apoptozole treatment eliminated the effects of AVA. Our study revealed that AVA improved colonic injury and inflammation induced by food allergies, and this mechanism may be mediated by the increased microbial-derived butyrate and involved in the Hsp70-NF-κB signaling.

## 1. Introduction

A Food allergy (FA) is classified as the adverse response toward food antigens mediated by the immune system, and it is a serious epidemic affecting people worldwide [1]. Milk, peanuts, soy, wheat, eggs, and shellfish are the major allergens to bring about food hypersensitivities [2]. The intestinal immune system can provide a balanced response to pathogens or food antigens, therefore preventing unnecessary inflammatory disorders and accompanied tissue damage [3]. The pathogenesis of FA is characterized by the induction of T helper 2 (Th2) cell reaction induced by the disruption of oral tolerance to exogenous allergens [4,5]. It is worth noting that the overexposure to food allergens can result in the intestinal inflammation and mucosal barrier injury, which thus makes it easier for food allergens to enter the bloodstream, and then aggravates the allergic reaction and intestinal damage [6,7]. Therefore, it is necessary to explore effective strategies to relieve intestinal injury and inflammation induced by food antigens.

Gut microbiota plays a part in multiple crucial physiological host functions [8], and has been suggested to have critical functions in protecting against allergic reactions to food antigens, indicating that the regulation of the bacterial community is a feasible strategy for the treatment of FA [9]. It is accepted that gut microbes benefit the host through synthesizing a great many beneficial metabolites, of which short-chain fatty acids (SCFAs) are essential for promoting intestinal functions [10]. The SCFAs produced in the colon principally include acetate, butyrate, and propionate. Butyrate is the main energy provider for the colon, and it also has a great impact on sustaining the intestinal wall integrity, preventing “leaky gut”, and regulating inflammatory response [11]. Furthermore, an in vivo study proved that butyrate markedly elevated the heat shock protein 70 (Hsp70) expression [12]. Hsp70 is known to be a representative member of the HSPs family, and its up-regulation can inhibit the generation of proinflammatory cytokines via blocking nuclear factor kappa-B (NF-κB) activation [13].

Polyphenols are a class of plant secondary metabolites that exert prebiotic-like activity by lowering the occurrence of disease [14]. Avenanthramides (AVAs) are soluble polyphenolic alkaloids unique to oats. Structurally, AVAs are conjugates of hydroxycinnamic acid or its derivatives and hydroxyanthranilic acid or its derivatives. AVAs can be extracted from oat grains and hulls with methanol or supercritical CO_2_ [15,16]. In addition, currently, there are more than 20 isoforms of AVAs identified in oat. It is found that AVA has a diversity of biological activities including anti-oxidative, anti-atherogenic and anti-inflammatory properties [17,18]. Our previous study revealed that AVA could attenuate the jejunal damage and allergic symptoms in mice induced by ovalbumin (OVA), and part of its regulatory mechanism was connected to the Hsp70-NF-κB signaling [19]. Notably, part of polyphenols act on and are absorbed in the small intestine, while most polyphenols reach the colon, which is the major colonization site of intestinal flora, and thus modulate the intestinal microbial structure [20]. Additionally, polyphenols have been confirmed to changing the SCFA synthesis in the intestine via regulating SCFA-producing microbiota composition [21], further implying their contribution to intestinal health. However, it is not clear that the effect of AVA on gut microbial alteration and whether the mechanism of AVA function in colonic damage induced by FA is related to the change in gut microbiota.

Based on the important findings mentioned above, this study was designed to probe into the efficiency of AVA on colonic inflammation and damage induced by FA and its mechanism. To this end, an OVA-induced FA mouse model was used throughout this study.

## 2. Materials and Methods

### 2.1. Animal Model Experiments

This study was conducted according to the animal protocol approved by the Institutional Animal Care and Use Committee of China Agricultural University (permission code: AW31402202-1-4). Six-week-old male BALB/c mice were employed in this study. Before experiments, mice were adapted the breeding environment for one week. Mice were allowed ad libitum access to water and feed, and were raised under a 12-h light/dark cycle system. In the breeding room, the temperature was kept at 22 °C, and the humidity was 55 ± 5%. 

In Exp. 1, to investigate the therapeutic effect of AVA on OVA-induced colonic injury and its appropriate dose, mice were randomly assigned into four groups (*n* = 10): the control group (CON), the OVA group, the OVA group with 10 mg/kg bw of AVA (OVA+LAVA), and the OVA group with 20 mg/kg bw of AVA (OVA+HAVA). Mice from the OVA, OVA+LAVA and OVA+HAVA groups were sensitized with 1 mg Alum adjuvant (Thermo Fisher, Waltham, MA, USA) and 50 μg OVA (Sigma-Aldrich, Madrid, Spain; purity ≥ 98.0%) in 200 µL saline by intraperitoneal injection twice at two-week intervals (day 0 and 14). During days 28~38, mice from these groups were challenged intragastrically with 50 mg OVA every other day for six times. Mice from the CON group were intraperitoneally sensitized and orally challenged with saline. Moreover, during days 0~38, mice from the CON group were intragastrically daily with saline, whereas mice from the AVA supplementation groups were intragastrically with AVA (Sigma-Aldrich, Madrid, Spain; purity ≥ 98.0%). On the sensitization or challenge days, the AVA administrations were conducted after 1 h of the OVA treatment.

Exp. 2 was designed to explore the effects of AVA on gut microbiota composition and to validate the contribution of Hsp70-NF-κB signaling to AVA function. Mice were randomly assigned into four groups (*n* = 10): the OVA group, the OVA+HAVA group, the OVA group combined with apoptozole (OVA+APO; Selleck, Houston, Texas, USA; purity ≥ 99.0%) and the OVA+HAVA group combined with apoptozole (OVA+HAVA+APO). All groups of mice were sensitized and challenged following the above procedures. Moreover, during days 0~38, mice from the high-dose AVA supplementation groups were intragastrically daily with AVA, whereas mice from the OVA and OVA+APO groups were intragastrically with saline at this point. Moreover, during days 0~38, mice from the two APO groups were intraperitoneally injected with APO dissolved in 4 mg/kg bw DMSO (20 µL) at intervals of 1 day [22]. The APO treatment was conducted 1 h prior to the sensitization or challenge, and mice from the OVA and OVA+HAVA groups received DMSO at the same time. 

After experiments, mice were euthanized after 12 h of fasting. The blood sample was rested for 30 min at room temperature, and the serum was attained after centrifugation from the blood sample under 4000× *g* for 10 min at 4 °C and then stored at −20 °C. The colon tissues and contents were collected and stored at liquid nitrogen for further analysis. 

### 2.2. Determination of Cytokines and Tight Junction Proteins

All indexes for ELISA were detected from the colonic tissue. The expressions of the cytokines including interferon-γ (IFN-γ), transforming growth factor-β (TGF-β), interleukin (IL)-4, IL-33, IL-13, IL-25, IL-10, tumor necrosis factor-α (TNF-α), and thymicstromal lymphopoietin (TSLP) were determined using by the commercial ELISA kits (eBioscience, San Diego, CA, USA). The level of slgA were also measured by ELISA kit (Bethyl Laboratories, Inc. Montgomery, TX, USA). The concentrations of tight junction proteins including claudin-1, occludin and ZO-1 were detected by the commercial ELISA kits (MyBioSource, San Diego, CA, USA).

### 2.3. Western Blot

Lysis buffer was used to extract total protein from colon tissues, and BCA Protein Assay Kit (Applygen Technologies Inc., Beijing, China) was put into use to detect protein concentrations. Primary antibodies including β-actin (1:1000), NF-κB (1:1000) and phosphorylated (*p*)-NF-κB (1:1000) were purchased from Cell Signaling Technology, Inc. (Danvers, MA, USA). In addition, the primary antibody of Hsp70 (1:1000) was obtained from Abcam (Cambridge, UK). Blots were incubated with horseradish peroxidase-coupled secondary antibody, and were finally quantified by ImageJ software (NIH, Bethesda, MD, USA).

### 2.4. Gut Microbiota Analysis

Methods for DNA extraction from colonic contents and the 16S rRNA gene sequencing were followed our previous work with modifications [23]. Generally, after extraction of the colonic microbiota genomes, PCR barcoded primers 338F (5′-ACTCCTACGGGAGGCAGCA-3′) and 806R (5′-GGACTACHVGGGTWTCTAAT-3′) were used to amplify the microbial 16S rRNA genes in the V3 and V4 regions. The amplified products were detected and purified with 2% agarose gel electrophoresis. Then, the purified PCR products were pooled for sequencing by the Illumina MiSeq platform (Majorbio, Shanghai, China). The open-source bioinformatics pipeline QIIME2 was used to process the raw sequences. The amplicon sequence variants (ASVs) were imported in QIIME2 to generate the taxonomy tables. The original data have been uploaded to the NCBI SRA Database (accession number: PRJNA904500).

### 2.5. Short-Chain Fatty Acid Detection

Colonic contents were collected, and then were homogenized in 15 mL of double-distilled water. The supernatants were obtained after centrifugating under 15,000× *g* for 15 min at 4 ℃, and were ulteriorly filtered through 0.22 µm pore. The analysis of SCFA was performed by gas chromatography on the basis of previous study [24]. Briefly, aliquots of 1 µL were injected into the capillary column. Both detector and injector temperatures were kept at 240 °C. The carrier gas was nitrogen and the flow rate was 2.0 mL/min.

### 2.6. Statistic Analysis

All data were analyzed using SPSS v. 22.0 (IBM Corp., Armonk, NY, USA). One-way ANOVA with Duncan’s multiple range test was used to detect cytokine concentrations, tight junction protein levels, SCFAs contents, and the protein gray value among different groups. The Kruskal–Wallis test was conducted to evaluate the abundance of gut microbiota. Spearman Rank analysis was conducted to analyze the association between the abundance of gut microbes and the concentrations of SCFAs. Data are presented as means ± SDs. Statistical significance was decided at *p* ≤ 0.05.

## 3. Results

### 3.1. Effect of AVA on the Colonic Barrier Damage Induced by FA in Exp. 1

**Figure 1:**
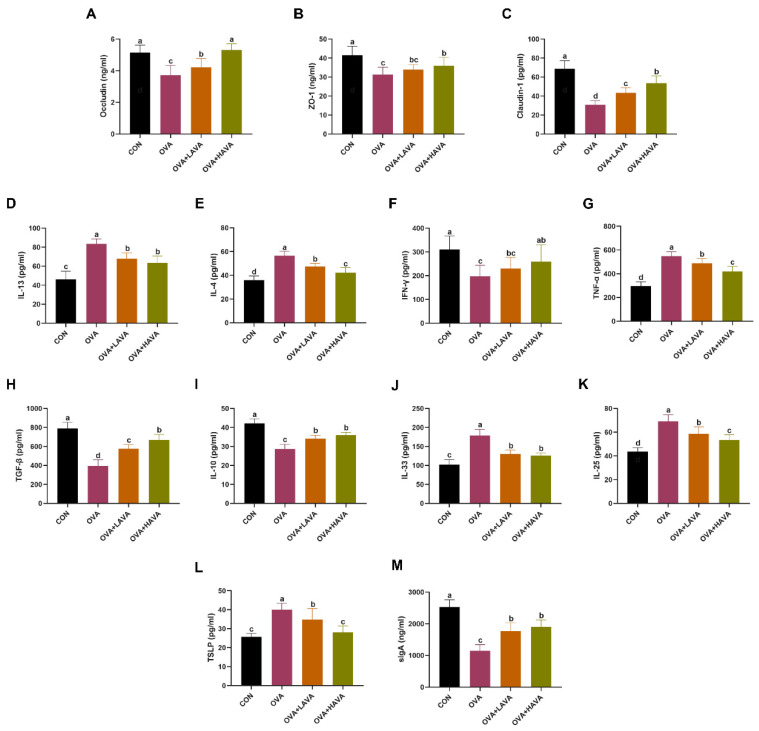
Effects of avenanthramide (AVA) on ovalbumin (OVA)-induced colonic barrier damage and allergic inflammation in mice in Exp. 1. (**A**–**C**) Levels of occludin, ZO-1 and claudin-1 of the colon, (**D**–**F**) levels of interleukin-13 (IL-13), IL-4 and interferon-γ (INF-γ) of the colon, (**G**–**I**) levels of tumor necrosis factor-α (TNF-α), transforming growth factor-β (TGF-β) and IL-10 of the colon, (**J**–**M**) levels of IL-33, IL-25, thymicstromal lymphopoietin (TSLP) and secretory IgA (sIgA) of the colon. CON: the control group; OVA: the group that challenged with OVA; OVA+LAVA: the OVA group that supplemented with 10 mg/kg bw AVA; OVA+HAVA: the OVA group that supplemented with 20 mg/kg bw AVA. Bars without the same letter are significantly different, *p* ≤ 0.05. Data are shown as means ± SDs (*n* = 9).

The concentrations of tight junction proteins in the colon are shown in Figure 1A–C. The colonic concentrations of occludin, ZO-1 and claudin-1 were observably lower in mice with OVA treatment than those in mice with CON treatment (*p* ≤ 0.05). In addition, markedly higher contents of occludin and claudin-1 were detected in both AVA gavaged treatments than in the OVA treatment (*p* ≤ 0.05), but lower contents of occludin and claudin-1 were found in the OVA+LAVA group than in the OVA+HAVA group (*p* ≤ 0.05). Moreover, the OVA+HAVA treatment visibly elevated the colonic expression of ZO-1 compared with the OVA treatment (*p* ≤ 0.05).

### 3.2. Effect of AVA on the Allergic Inflammatory Response of Colon in Exp. 1

Indicators about the allergic inflammatory response in the colon are shown in Figure 1D–M. By compared with mice from the other treatments, mice from the OVA treatment had elevated concentrations of Th2 cytokines including IL-13 and IL-4 (*p* ≤ 0.05). In addition, IL-4 was lower in the OVA+HAVA treatment compared to that in the OVA+LAVA treatment (*p* ≤ 0.05). Moreover, mice in the OVA treatment had a reduced concentration of Th1 cytokine IFN-γ when compared to mice in the CON and OVA+HAVA treatments (*p* ≤ 0.05; Figure 1D–F). Moreover, the level of pro-inflammatory cytokine TNF-α increased in the colon of mice by the OVA treatment relative to mice by the other treatments (*p* ≤ 0.05), and decreased in the colon of mice by the OVA+HAVA treatment relative to mice by the OVA+LAVA treatment (*p* ≤ 0.05; Figure 1G). Additionally, the concentrations of anti-inflammatory factors including TGF-β and IL-10 were remarkably lower in the colon of mice by the OVA treatment in comparison to the other treatments (*p* ≤ 0.05), and TGF-β in the colon was higher in the OVA+HAVA group compared to the OVA+LAVA group (*p* ≤ 0.05; Figure 1H,I). Moreover, in the colon, mice of the OVA group displayed the markedly enhanced expressions of IL-33, IL-25 and TSLP compared with mice of the other groups (*p* ≤ 0.05), and the remarkably declined contents of TSLP and IL-25 were determined in the OVA+HAVA treatment in contrast to the OVA+LAVA treatment (*p* ≤ 0.05; Figure 1J–L). Mice in the OVA treatment showed the decreased level of sIgA than the CON group (*p* ≤ 0.05), but mice in the two AVA treatments had the higher sIgA level than the OVA group (*p* ≤ 0.05; Figure 1M).

### 3.3. Effect of AVA on the Hsp70-NF-kB Signaling in Exp. 1

**Figure 2:**
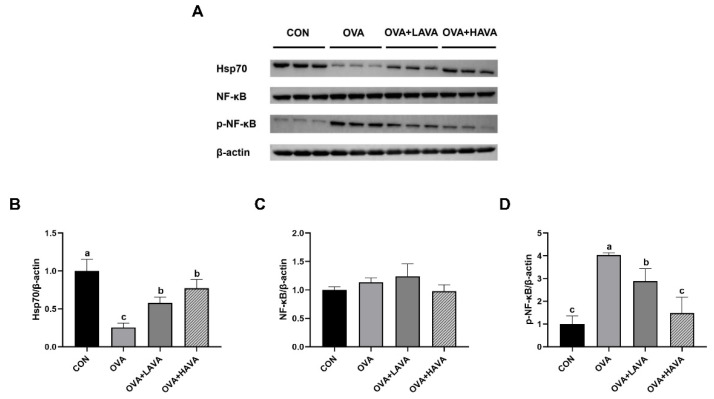
Effects of avenanthramide (AVA) on the colonic expressions of heat shock protein 70 (Hsp70) and nuclear factor kappa-B (NF-κB) in ovalbumin (OVA)-induced food allergy mice in Exp. 1. (**A**) Protein expressions of β-actin, Hsp70, NF-κB and phosphorylation of NF-κB (*p*-NF-κB) of the colon, (**B**–**D**) proteins quantification of Hsp70, NF-κB and *p*-NF-κB of the colon. CON: the control group; OVA: the group that challenged with OVA; OVA+LAVA: the OVA group that supplemented with 10 mg/kg bw AVA; OVA+HAVA: the OVA group that supplemented with 20 mg/kg bw AVA. Bars without the same letter are significantly different, *p* ≤ 0.05. Data are shown as means ± SDs (*n* = 3).

The results on the AVA effect on Hsp70-NF-kB signaling are presented in Figure 2. In Exp. 1, as shown in Figure 2A, no changes in β-actin and NF-kB were observed among the groups. Importantly, the protein level of Hsp70 in the colon of mice from the OVA treatment was markedly lower than that of mice from the other treatments (*p* ≤ 0.05). However, both AVA supplementation groups showed the increased Hsp70 expression relative to the OVA group (*p* ≤ 0.05). Consistently, the protein content of *p*-NF-κB in the colon increased in mice of the OVA group in contrast with that in mice of the other groups (*p* ≤ 0.05), and decreased in mice with the high dose of AVA ingestion compared with that in mice with the low dose of AVA ingestion (*p* ≤ 0.05).

### 3.4. Effect of Hsp70 Inhibition on the Role of AVA in Colonic Damage Induced by OVA in Exp. 2

**Figure 3:**
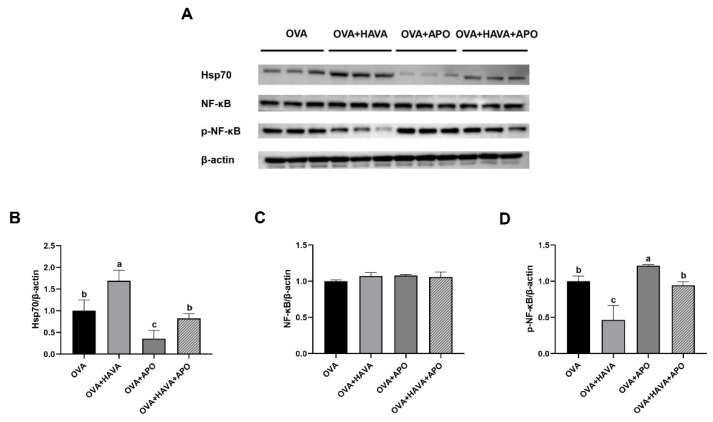
Effects of avenanthramide (AVA) on the colonic expressions of heat shock protein 70 (Hsp70) and nuclear factor kappa-B (NF-κB) in ovalbumin (OVA)-induced food allergy mice in Exp. 2. (**A**) Protein expressions of Hsp70, NF-κB and phosphorylation of NF-κB (*p*-NF-κB) of the colon, (**B**–**D**) proteins quantification of Hsp70, NF-κB and *p*-NF-κB of the colon. OVA: the group that challenged with OVA; OVA+HAVA: the OVA group that supplemented with 20 mg/kg bw AVA; OVA+APO: the OVA group that combined with apoptozole; OVA+HAVA+APO: the OVA+HAVA group that combined with apoptozole. Bars without the same letter are significantly different, *p* ≤ 0.05. Data are shown as means ± SDs (*n* = 3).

Based on the above findings, we speculated that AVA attenuated colonic damage induced by OVA through regulating the Hsp70-NF-κB signaling. Therefore, in Exp. 2, apoptozole, which is a known Hsp70 inhibitor, was employed to verify the Hsp70-NF-κB signaling contribution to the beneficial function of AVA. As exhibited in Figure 3, the Hsp70 expression was elevated in the colon of mice by OVA+HAVA treatment relative to mice by the other treatments (*p* ≤ 0.05), and was declined in the colon of mice treated by the OVA+APO compared with mice treated by the OVA+HAVA+APO and OVA (*p* ≤ 0.05). Furthermore, in the colon, the *p*-NF-κB expression was lower in the OVA+HAVA treatment than that in the other treatments (*p* ≤ 0.05) and was higher in OVA+APO treatment than that in the OVA+HAVA+APO and OVA treatments (*p* ≤ 0.05). No significant differences in the protein levels of Hsp70 and *p*-NF-κB were found between the OVA and OVA+HAVA+APO groups (*p* > 0.05).

**Figure 4:**
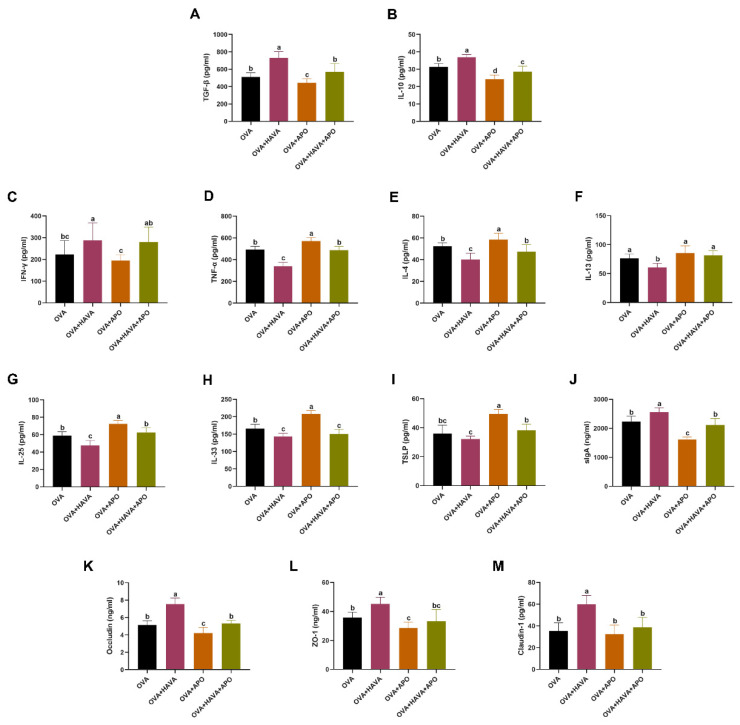
Effects of avenanthramide (AVA) on ovalbumin (OVA)-induced colonic allergic inflammation and barrier damage in mice in Exp. 2. (**A**–**C**) Levels of transforming growth factor-β (TGF-β), interleukin-10 (IL-10) and interferon-γ (INF-γ) of the colon, (**D**–**F**) levels of tumor necrosis factor-α (TNF-α), IL-4 and IL-13 of the colon, (**G**–**J**) levels of IL-25, IL-33, thymicstromal lymphopoietin (TSLP) and secretory IgA (sIgA) of the colon, (**K**–**M**) levels of occludin, ZO-1 and claudin-1 of the colon. OVA: the group that challenged with OVA; OVA+HAVA: the OVA group that supplemented with 20 mg/kg bw AVA; OVA+APO: the OVA group that combined with apoptozole; OVA+HAVA+APO: the OVA+HAVA group that combined with apoptozole. Bars without the same letter are significantly different, *p* ≤ 0.05. Data are shown as means ± SDs (*n* = 9).

As shown in Figure 4A–J, with Hsp70 inhibition, the effects of AVA on colonic inflammation induced by OVA were determined. In the colon, the expressions of TGF-β and IL-10 were elevated in mice of the OVA+HAVA treatment compared with mice of the other treatments (*p* ≤ 0.05), and decreased in mice of the OVA+APO treatment relative to that in mice of the OVA+HAVA+APO and OVA treatments (*p* ≤ 0.05). In addition, the content of IFN-γ was lower in the colon of mice by OVA+APO treatment than that of mice by OVA+HAVA and OVA+HAVA+APO treatments (*p* ≤ 0.05), and the IFN-γ level had no difference between the OVA and OVA+HAVA+APO treatments (*p* > 0.05; Figure 4A–C). The colonic contents of TNF-α, IL-4 and IL-13 were reduced in the OVA+HAVA treatment relative to those in the other treatments (*p* ≤ 0.05), but there was no significant difference in the colonic contents of TNF-α, IL-4 and IL-13 between the OVA and OVA+HAVA+APO treatments (*p* > 0.05; Figure 4D–F). Moreover, the levels of IL-25 and IL-33 were lower in the colon of mice by OVA+HAVA treatment than those of mice by OVA and OVA+APO treatments (*p* ≤ 0.05), and the colon level of TSLP was lower in the OVA+HAVA treatment than that in the OVA+APO and OVA+HAVA+APO treatments (*p* ≤ 0.05). The contents of IL-25, IL-33, and TSLP were all remarkably higher in the colon of mice by OVA+APO treatment than those of mice by other treatments (*p* ≤ 0.05). Furthermore, the OVA+HAVA treatment increased the concentration of sIgA relative to the other groups (*p* ≤ 0.05), and the OVA+APO treatment decreased the concentration of sIgA relative to the OVA+HAVA+APO and OVA treatments (*p* ≤ 0.05). No difference in sIgA level was determined between the OVA and OVA+HAVA+APO treatments (*p* > 0.05; Figure 4G–J). 

The results about the effect of AVA on OVA-induced colonic barrier injury under Hsp70 activity inhibition are shown in Figure 4K–M. It was found that the levels of occludin, ZO-1 and claudin-1 in the colon were evidently elevated in mice of OVA+HAVA treatment relative to mice of other treatments (*p* ≤ 0.05). Moreover, no significant difference was observed in the levels of occludin, ZO-1 and claudin-1 between the OVA and OVA+HAVA+APO treatments (*p* > 0.05).

### 3.5. Effect of AVA on Alteration of Gut Microbes in Exp. 2

**Figure 5:**
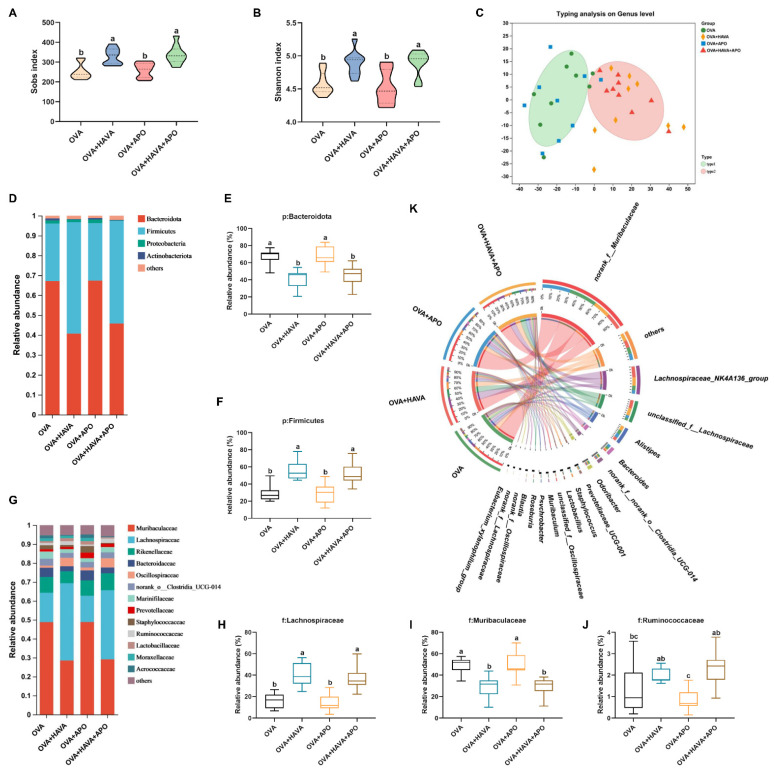
Avenanthramide (AVA) altered the colonic microbiota composition in ovalbumin (OVA)-induced food allergy mice in Exp. 2. (**A**–**B**) Alpha diversity with the Sobs index and the Shannon index of the colonic microbiota, (**C**) beta diversity with the enterotype analysis of the colonic microbiota on the genus level, (**D**–**F**) the relative abundance of the colonic microbiota on phylum level, (**G**–**J**) the relative abundance of the colonic microbiota on family level, (**K**) the chord diagram showing the relative abundance of the colonic microbial community on the genus level. OVA: the group that challenged with OVA; OVA+HAVA: the OVA group that supplemented with 20 mg/kg bw AVA; OVA+APO: the OVA group that combined with apoptozole; OVA+HAVA+APO: the OVA+HAVA group that combined with apoptozole. Bars without the same letter are significantly different, *p* ≤ 0.05. Data are shown as means ± SDs (*n* = 9).

In Exp. 2, 16S rRNA sequencing was conducted to figure out whether AVA makes a difference in the colonic microorganism structure. Alpha diversity analysis with the Sobs index and the Shannon index revealed that microbial richness and diversity were both increased in the two AVA consumption treatments in contrast to the OVA and OVA+APO treatments (*p* ≤ 0.05; Figure 5A,B). In addition, enterotype analysis on the genus level displayed that the microbial communities of the two AVA administration groups were distinct from those of the other two groups, suggesting that AVA could markedly alter the colonic microbial composition (Figure 5C).

Within the phylum level, the relative abundance of Bacteroidota decreased, while the relative abundance of Firmicutes was enhanced in the colon of mice treated by AVA administrations in comparison with mice in the OVA+APO and OVA treatments (*p* ≤ 0.05; Figure 5D–F and Table 1). Additionally, the OVA+HAVA group presented a reduced abundance of Actinobacteriota compared to the OVA group (*p* ≤ 0.05; Table 1). Within the family level, AVA administrations significantly raised the relative abundance of Lachnospiraceae and Oscillospiraceae (*p* ≤ 0.05), but lowered the relative abundance of Muribaculaceae relative to the OVA+APO and OVA treatments (*p* ≤ 0.05; Figure 5G–I and Table 1). Moreover, Ruminococcaceae was higher in the colon of mice in the OVA+HAVA and OVA+HAVA+APO groups than that of mice treated by the OVA+APO (*p* ≤ 0.05; Figure 5J and Table 1). Butyricicoccaceae was higher in the colon of mice in the OVA+HAVA+APO group than that of mice in the OVA+APO and OVA groups (*p* ≤ 0.05; Table 1). Moreover, the OVA+HAVA treatment exhibited a lower proportion of Bacteroidaceae than the OVA+APO treatment (*p* ≤ 0.05; Table 1). Within the genus level, the OVA+APO and OVA groups showed a lower abundance of *Lachnospiraceae_NK4A136_group* than the OVA+HAVA group (*p* ≤ 0.05), and a lower abundance of *Roseburia* than the OVA+HAVA+APO group (*p* ≤ 0.05). Mice in the OVA treatment had a declined level of *Blautia* realtive to the mice in the OVA+HAVA+APO and OVA+HAVA treatments (*p* ≤ 0.05). Additionally, in contrast to the other groups, the AVA ingestion groups both significantly decreased the portion of *norank_f_Muribaculaceae* while increased the amount of *unclassified_f_Lachnospiraceae* (*p* ≤ 0.05; Figure 5K and Table 1).

### 3.6. Effect of AVA on Short-Chain Fatty Acids Content in Exp. 2

**Figure 6:**
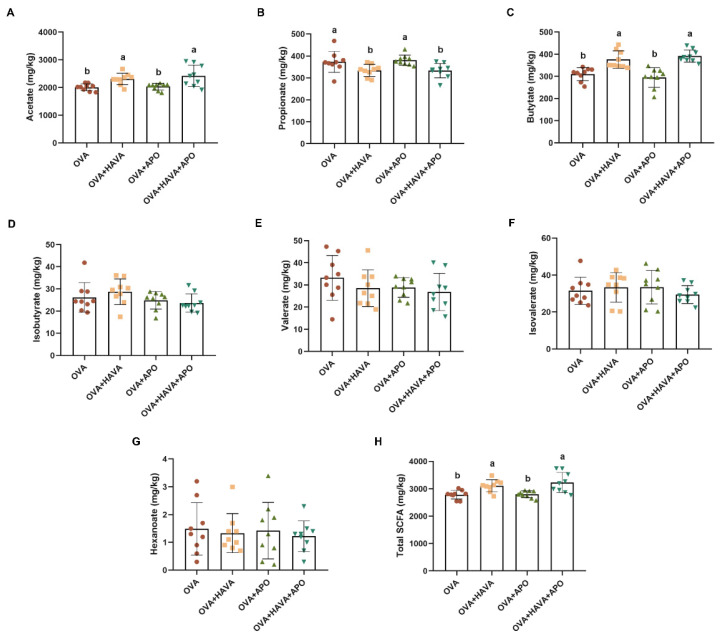
Avenanthramide (AVA) changed the short-chain fatty acids (SCFAs) contents of the colon in ovalbumin (OVA)-induced food allergy mice in Exp. 2. (**A**–**C**) Concentrations of acetate, propionate, and butyrate of the colon, (**D**–**G**) concentrations of isobutyrate, valerate, isovalerate and hexanoate of the colon, (**H**) concentrations of total short-chain fatty acids of the colon. OVA: the group that challenged with OVA; OVA+HAVA: the OVA group that supplemented with AVA (20 mg/kg bw); OVA+APO: the OVA group that combined with apoptozole; OVA+HAVA+APO: the OVA+HAVA group that combined with apoptozole. Bars without the same letter are significantly different, *p* ≤ 0.05. Data are shown as means ± SDs (*n* = 9).

Figure 6 showed different concentrations of SCFAs in the colonic contents of mice. The concentrations of acetate and butyrate were remarkably elevated in the AVA addition groups relative to the OVA+APO and OVA groups (*p* ≤ 0.05). However, a significant decline of propionate was found in both AVA consumption groups in contrast to the OVA and OVA+APO groups (*p* ≤ 0.05). The levels of isobutyrate, valerate, isovalerate, and hexanoate had no alteration among all groups (*p* > 0.05). In addition, the OVA and the OVA+APO treatments had reduced contents of total SCFAs than the two AVA treatments (*p* ≤ 0.05).

### 3.7. Correlation between Gut Microbes and Short-Chain Fatty Acids in Exp. 2

**Figure 7:**
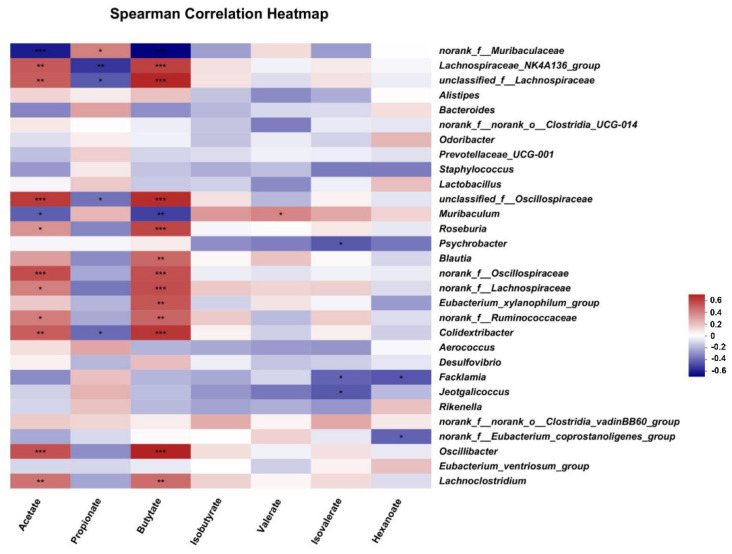
The Spearman analysis of the correlation between colonic microbes and short-chain fatty acids in Exp. 2. *** *p* ≤ 0.001, ** 0.001 < *p* ≤ 0.01, * 0.01 < *p* ≤ 0.05.

To identify the association between gut microbes at genus level and SCFAs, the Spearman correlation analysis was preformed (Figure 7). The abundance of *norank_f_Muribaculaceae* was negatively associated with the concentrations of acetate and butyrate (*p* ≤ 0.05), but was positively related to the level of propionate (*p* ≤ 0.05). Moreover, the abundance of *Lachnospiraceae_NK4A136_group* was negatively related to the level of propionate (*p* ≤ 0.05), while was positively correlated with the levels of butyrate and acetate (*p* ≤ 0.05). In addition, acetate and butyrate also showed remarkably positive correlations with the abundance of *Colidextribacter*, *Oscillibacter* and *Lachnoclostridium* (*p* ≤ 0.05). Moreover, *Roseburia*, *norank_f_Lachnospiraceae* and *norank_f_Ruminococcaceae* abundance had positive correlations with acetate and butyrate concentrations (*p* ≤ 0.05). Furthermore, the abundance of *Blautia* showed a positive association with the concentration of butyrate (*p* ≤ 0.05).

## 4. Discussion

Food allergens can result in increased gut permeability and induce gut inflammation. Once the intestine is damaged, allergens will enter the peripheral circulation more smoothly, and will further intensify the allergic response [6,7]. In this study, we explored the efficacy of oral AVA on OVA-induced colonic injury in mice, and inquired about the possible mechanism. Our study showed that AVA had an alleviating effect on colonic damage in FA mouse model, which might be realized by modulating gut microbial structure and SCFAs compositions, especially up-regulating the abundance of butyrate-producing microbes as well as the level of butyrate, and subsequently promoting Hsp70 expression and inhibiting NF-κB activation in the colon.

AVA as a kind of phenolic compound was reported to mediate anti-inflammatory activities in neuron and derma, and corresponding mechanism of which was related to the reduced expression of pro-inflammatory factors and elevated production of anti-inflammatory factors in tissues [25]. It is reported that cytokines including IL-25, TSLP and IL-33 secreted by damaged or stressed intestinal epithelium promote the development of Th2 immune responses and thus exacerbate food allergic responses [26]. In Exp. 1 of this study, OVA-induced mice did have significantly increased productions of IL-25, TSLP and IL-33 in the colon in contrast with the mice in the CON treatment. However, mice receiving additional AVA showed the markedly decreased colonic contents of TSLP, IL-33 and IL-25 compared with mice treated with OVA alone. These findings were consistent with the protective function of AVA against chronic inflammatory processes related to allergic diseases [25,27]. Th1 cells secrete large amounts of IFN-γ to protect the organism from intracellular pathogens [28], whereas Th2 cells mainly release cytokines including IL-13, IL-4 and IL-5 that are able to reinforce humoral immunity [29]. Under normal circumstances, Th1 and Th2 cells coordinate with each other to achieve dynamic balance through the action of cytokines, and receive the regulation of helper T cells at the same time [28,29]. When the balance is broken by external allergens, the Th2 cell is over-activated, resulting in the occurrence of an allergy in the body, accompanied by symptoms of tissue inflammation [30,31]. In Exp. 1, both AVA treatments decreased the colonic contents of IL-4 and IL-13, and the high dose of AVA supplementation increased the level of IFN-γ. These results suggested that AVA could suppress the OVA-induced allergic inflammation reactions via adjusting the Th1/Th2 imbalance. 

The IL-10 and TGF-β are anti-inflammatory factors engaged in maintaining intestinal mucosal homeostasis by controlling the Th2 response, thus inhibiting the allergic response and intestinal inflammatory progression [28,32]. Previous research suggested that the elevation of TGF-β and the reduction of TNF-α prevented the damage to the intestinal barrier in food allergic animal models [33,34]. Furthermore, TGF-β can facilitate IgA synthesis, and IgA is considered to be a pivotal mucosal antibody with a significantly defensive property [35,36]. In Exp. 1, the colonic concentrations of IL-10 and TGF-β increased in both AVA intake groups in contrast to the OVA group, accompanied by an elevated sIgA concentration and depressed TNF-α content in the colon. These findings further revealed the beneficial efficiency of AVA on alleviating FA-induced colonic inflammation and indicated a possible improvement of the intestinal barrier by AVA. Under inflammatory conditions, the expressions of tight junction proteins are inhibited and thus the gut permeability increases, exacerbating the inflammatory response [6,37]. In view of the results of Exp. 1, the concentrations of tight junction proteins such as occludin, claudin-1 and ZO-1 in the colon of AVA administration mice were enhanced in contrast to those in the OVA group, which verified that OVA exposure damaged epithelial barrier integrity, while AVA attenuated this process and preserved the intestinal barrier.

The gastrointestinal tract harbors numerous microbes, whose composition and diversity are directly associated with host health [38]. Gut microbial metabolites, such as SCFAs, are vital signals that mediate complicated interactions between the host and gut microbiome. SCFAs are regulated by alterations in the composition of gut microbes [39]. Acetate, butyrate, and propionate are the major SCFA produced from the dietary carbohydrate fermentation and generated from different synthetic pathways [40]. In the gut, propionate can be synthesized from phosphoenolpyruvate through the propanediol and the succinate pathways, but acetate is primarily produced from pyruvate via the acetyl-CoA pathway. Moreover, the butyryl CoA:acetate-CoA-transferase route is a main process to form butyrate, in which butyrate producers use acetate to synthesize butyrate in the colonic ecosystem [40]. In this present study, the contents of acetate and butyrate were elevated while the concentration of propionate was reduced after AVA supplementation. Since the main mechanism of altering SCFA composition could involve changes in gut microbiota, these results might be partly attributed to a great improvement on the abundance of acetate- or butyrate-producing bacteria induced by AVA, and subsequently these bacteria compete for fermented substrates, thus inhibiting the propionate production. In this study, the dramatically lower abundance of Bacteroidota and Muribaculaceae in AVA-treated mice might contribute to the decreased propionate. Dietary carbohydrates driven by the Bacteroidota are the primary route for propionate generation, and the relative abundance of Bacteroidota was demonstrated to correlate with the content of fecal propionate in humans [41]. Additionally, although few isolates are available, the bacterial family Muribaculaceae are plentiful and diverse in the mice gut, and its members have been shown to produce propionate as a major fermentation product [42]. Consistent with our results, the abundance of Muribaculaceae was demonstrated to be bound up with propionate levels [43], further favoring the assumption that Muribaculaceae might be a principal degradation of some polysaccharides and a producer of propionate. Moreover, our results indicated that the additional AVA consumption expanded the portion of Lachnospiraceae and Ruminococcaceae in the colon of mice, which are thought to be protective microbes rich in the gut of humans and other mammals because of their ability to produce butyrate [44]. Within the Lachnospiraceae family, *Roseburia* and *Blautia* are genera that have been shown to be closely involved in the process of reliving intestinal inflammation, manifested by enhanced production of anti-inflammatory cytokines (e.g., TGF-β and IL-10) in the gut, and these underlying mechanisms are mediated by the end products of their bacterial metabolism, particularly butyrate [45]. In addition, the genus *NK4A136 group*, a member belonging to the Lachnospiraceae family, is commonly connected with intestinal mucosal barrier repair and the improvement in colitis, and these effects may be due to its butyrate production capacity to provide benefits for the gut in terms of immune regulation [46]. In the study, our results revealed that the AVA supplementation regulated microbial composition, increasing the genera abundance of *norank_f_Ruminococcaceae*, *Roseburia*, *Blautia,* and *Lachnospiraceae_NK4A136_group*, which might result in the higher concentration of butyrate, and thus improved intestinal barrier and attenuated colonic inflammation.

AVA is known to be structurally similar to the synthetic oral drug Tranilast [18]. As an antiallergic agent, Tranilast can suppress NF-κB activation and has also been applied to treat inflammatory diseases [47]. NF-κB is identified as a transcriptional mediator that regulates the expressions of various genes implicated in inflammation and immunity, such as pro-inflammatory factors and immune receptors [48]. HSPs are a group of extremely conserved proteins existing from microorganisms to mammals, and in experimental disease models, it has been found that HSP administration could arrest inflammatory damage [49]. Hsp70 is the most famous member of the HSPs family, and it is reported that overexpression of Hsp70 could reduce the production of pro-inflammatory factors by blocking NF-κB activation, therefore achieving an anti-inflammatory state [13]. Additionally, the intestinal Hsp70 has been demonstrated to play a significant part in protecting epithelial cells in the intestine from stress or injury [50]. In Exp. 1 of this study, the protein expression of the colonic Hsp70 increased in the AVA ingestion groups more than that in the OVA treatment, along with blocked activation of NF-κB and reduced productions of pro-inflammatory cytokines. These results indicated that the Hsp70-NF-κB signaling was probably implicated in AVA amelioration of allergy-induced intestinal injury.

To validate the action of Hsp70 in AVA relieving intestinal damage induced by FA, AVA combined with a Hsp70 inhibitor was adopted to treat OVA-induced FA mice in Exp. 2. In an in vitro experiment, apoptozole as the Hsp70 inhibitor was found to suppress the ATPase activity of Hsp70 while did not affect the Hsp70 expression [22]. Nevertheless, in our study, we observed that the colonic Hsp70 expression in mice exposed to apoptozole was significantly declined, which possibly was due to differences at the tissue and cellular levels. In Exp. 2, the down-regulated and inhibited Hsp70 subsequently resulted in the increased NF-κB activation. Moreover, we identified that the antagonism of apoptozole to intestinal Hsp70 attenuated the anti-allergic effects of AVA. However, compared with the OVA+HAVA group, the OVA+HAVA+APO group did not entirely abolish the AVA effects, so we suppose that AVA acts not only via the Hsp70-NF-κB signaling, but more mechanisms still need to be disclosed. Interestingly, although Hsp70 activation has been proposed to protect the intestinal epithelial from damage [51], the role of Hsp70 in inflammatory responses remains unclear. Some other studies suggested that the activation of heat shock response had an anti-inflammatory function prior to injury, whereas a pro-inflammatory stimulus induced by heat shock response exerted under injury [52,53]. Therefore, the relationship between heat shock response and the point in time of action still should be further explored. In short, our findings further highlighted that the beneficial function of AVA on colonic injury induced by FA might be partially attributed to the enhanced Hsp70 expression and the blocked NF-κB activation in the intestine. 

It is worth noting that butyrate has been proved to reduce the action of NF-κB and suppress the generation of inflammatory factors, therefore mitigating the local inflammatory reaction in the intestine [54]. Another study reported that physiological concentrations of butyrate increased the expressions of gut tight junction proteins through inhibiting NF-κB signaling, and had a significantly promoting effect on the epithelial barrier of gut [55]. Additionally, previous research suggested that butyrate can promote Hsp70 expression and thus play an anti-inflammatory role [12,56]. Therefore, we assumed that the greater amount of butyrate upon AVA treatments may be related to the protective function of AVA in alleviating OVA-induced colonic injury. In Exp. 2, a validation group using food allergic mice treated by AVA and apoptozole was conducted, and in this group, the butyrate content was elevated but the beneficial effect of AVA on colon was weaker to an extent. In this study, these findings provided supporting evidence that the increase in butyrate by AVA administration might be helpful to lightening colonic injury induced by FA through the Hsp70-NF-κB signaling.

Our previous study found that AVA could relieve OVA-induced jejunal injury, and the Hsp70-NF-κB signaling was partially involved in the AVA function [19]. An in vitro cellular assay showed that Tranilast reduced LPS-induced secretion of inflammatory factors by blocking the CXCR4/JAK2/STAT3 signaling [57]. Therefore, we speculate that the role of AVA in the small intestine may be more similar to the pharmacological activity of the oral drug Tranilast, and AVA might directly target the Hsp70-NF-kB signaling in the small intestine of FA mice. Notably, it was reported that most of the total polyphenol intake accumulate in the colon and are metabolized by the colonic microbiota, and the polyphenols in turn modulate the composition of intestinal bacteria [58]. The number of bacteria varies in different parts of the gastrointestinal tract, and only a small number of bacteria reside in the stomach and upper part of the small intestine, while the colon is the most abundant in bacteria [59]. Indeed, in the present study, we found that AVA expanded the abundance of butyrate-producing bacteria in the colon of FA mice, and that the increased butyrate might subsequently up-regulate the expression of Hsp70 and inhibit the activation of NF-κB. Collectively, in this study, our results in the colon of FA mice further uncovered the relationship between AVA function and intestinal microbes, but the mechanism of AVA action still needs to be explored in depth.

## 5. Conclusions

In summary, our study suggested that the administration of AVA played a role in improving colonic inflammation and barrier function in mice induced by OVA. In addition, AVA visibly regulated the microbial community structure and altered the SCFA compositions, especially enhanced the level of butyrate. Our findings revealed that the potential mechanism of AVA function was partially implicated in Hsp70-NF-κB signaling, specifically an activation of Hsp70 and an inhibition of NF-κB, and this mechanism might be mediated by the increased microbial-derived butyrate. Our study provides a new perspective on how to mitigate intestinal injury in the context of food allergies.

## Figures and Tables

**Table 1:** The relative abundance of the top 30 microbiomes of the colonic microbiota in mice in Exp. 2.

Variable	OVA	OVA+HAVA	OVA+APO	OVA+HAVA +APO	*p* Value
Bacteroidota	67.1 ± 8.41 ^a^	41.2 ± 11.0 ^b^	67.7 ± 11.2 ^a^	45.6 ± 11.7 ^b^	<0.01
Firmicutes	28.9 ± 9.00 ^b^	55.5 ± 11.5 ^a^	28.7 ± 11.6 ^b^	51.8 ± 12.2 ^a^	<0.01
Desulfobacterota	0.42 ± 0.26	0.89 ± 0.53	0.30 ± 0.30	0.72 ± 0.58	0.05
Actinobacteriota	1.03 ± 0.98 ^a^	0.26 ± 0.20 ^b^	0.78 ± 0.65 ^ab^	0.31 ± 0.19 ^ab^	0.02
Muribaculaceae	49.2 ± 7.22 ^a^	29.0 ± 9.87 ^b^	49.5 ± 12.4 ^a^	29.1 ± 8.18 ^b^	<0.01
Lachnospiraceae	15.9 ± 6.95 ^b^	40.8 ± 11.00 ^a^	13.8 ± 7.63 ^b^	36.8 ± 10.9 ^a^	<0.01
Bacteroidaceae	4.62 ± 2.64 ^ab^	2.59 ± 1.37 ^b^	5.21 ± 2.37 ^a^	3.06 ± 1.28 ^ab^	0.04
Oscillospiraceae	1.13 ± 0.63 ^b^	4.39 ± 3.24 ^a^	1.50 ± 0.83 ^b^	4.87 ± 2.58 ^a^	<0.01
Ruminococcaceae	1.29 ± 1.11 ^bc^	1.99 ± 0.34 ^ab^	0.86 ± 0.49 ^c^	2.32 ± 0.79 ^ab^	<0.01
Desulfovibrionaceae	0.42 ± 0.26 ^b^	0.89 ± 0.53 ^a^	0.30 ± 0.30 ^b^	0.72 ± 0.58 ^ab^	0.05
Corynebacteriaceae	0.59 ± 0.91	0.04 ± 0.02	0.53 ± 0.56	0.09 ± 0.12	0.09
Peptococcaceae	0.16 ± 0.09 ^b^	0.28 ± 0.16 ^ab^	0.18 ± 0.13 ^b^	0.43 ± 0.15 ^a^	<0.01
Anaerovoracaceae	0.12 ± 0.09 ^ab^	0.14 ± 0.05 ^ab^	0.07 ± 0.05 ^b^	0.19 ± 0.07 ^a^	<0.01
Butyricicoccaceae	0.04 ± 0.08 ^b^	0.17 ± 0.13 ^ab^	0.05 ± 0.06 ^b^	0.22 ± 0.15 ^a^	<0.01
*norank_f__Muribaculaceae*	47.5 ± 7.21 ^a^	28.4 ± 9.56 ^b^	48.0 ± 11.7 ^a^	28.4 ± 7.93 ^b^	<0.01
*Lachnospiraceae_NK4A136_group*	8.48 ± 4.47 ^bc^	19.0 ± 5.03 ^a^	5.84 ± 3.14 ^c^	15.0 ± 7.39 ^ab^	<0.01
*unclassified_f_Lachnospiraceae*	4.62 ± 1.86 ^b^	14.5 ± 4.78 ^a^	4.91 ± 3.19 ^b^	13.7 ± 5.36 ^a^	<0.01
*Bacteroides*	4.62 ± 2.64 ^ab^	2.59 ± 1.37 ^b^	5.21 ± 2.37 ^a^	3.06 ± 1.28 ^ab^	0.05
*unclassified_f_Oscillospiraceae*	0.58 ± 0.34 ^b^	1.56 ± 0.70 ^a^	0.75 ± 0.37 ^b^	1.52 ± 0.50 ^a^	<0.01
*Muribaculum*	1.69 ± 0.80 ^a^	0.63 ± 0.58 ^b^	1.43 ± 1.24 ^ab^	0.63 ± 0.42 ^b^	0.01
*Roseburia*	0.31 ± 0.32 ^d^	1.37 ± 0.86 ^bc^	0.54 ± 0.62 ^cd^	1.77 ± 0.86 ^ab^	<0.01
*Blautia*	0.44 ± 0.28 ^b^	1.14 ± 0.59 ^a^	0.56 ± 0.52 ^ab^	1.70 ± 1.37 ^a^	<0.01
*norank_f_Oscillospiraceae*	0.20 ± 0.17 ^b^	1.18 ± 1.53 ^ab^	0.30 ± 0.21 ^b^	1.38 ± 1.22 ^a^	<0.01
*norank_f_Lachnospiraceae*	0.26 ± 0.29 ^b^	1.45 ± 1.46 ^a^	0.18 ± 0.18 ^b^	0.97 ± 0.65 ^a^	<0.01
*Eubacterium_xylanophilum_group*	0.33 ± 0.31 ^cd^	0.59 ± 0.53 ^bc^	0.53 ± 0.57 ^bc^	1.27 ± 0.75 ^ab^	0.02
*norank_f_Ruminococcaceae*	0.54 ± 0.64 ^ab^	0.68 ± 0.18 ^a^	0.24 ± 0.14 ^b^	0.76 ± 0.48 ^a^	<0.01
*Colidextribacter*	0.15 ± 0.11 ^c^	0.75 ± 0.49 ^ab^	0.22 ± 0.17 ^bc^	0.98 ± 0.50 ^a^	<0.01
*Rikenella*	0.57 ± 0.29 ^a^	0.27 ± 0.10 ^b^	0.51 ± 0.28 ^ab^	0.36 ± 0.18 ^ab^	0.01
*Lachnoclostridium*	0.25 ± 0.11 ^ab^	0.47 ± 0.27 ^ab^	0.20 ± 0.12 ^b^	0.49 ± 0.23 ^a^	0.01
*Oscillibacter*	0.08 ± 0.09 ^b^	0.56 ± 0.49 ^a^	0.10 ± 0.10 ^b^	0.65 ± 0.54 ^a^	<0.01

^a–d^ There were no significant differences in means with the same letter in each row. OVA: the group that challenged with OVA; OVA+HAVA: the OVA group that supplemented with AVA (20 mg/kg bw); OVA+APO: the OVA group that combined with apoptozole; OVA+HAVA+APO: the OVA+HAVA group that combined with apoptozole. Data are shown as means ± SDs (*n* = 9).
